# Portfolio Tail Risk: A Multivariate Extreme Value Theory Approach

**DOI:** 10.3390/e22121425

**Published:** 2020-12-17

**Authors:** Miloš Božović

**Affiliations:** Faculty of Economics, University of Belgrade, Kamenička 6, 11000 Belgrade, Serbia; milos.bozovic@ekof.bg.ac.rs

**Keywords:** tail risk, extreme value theory, principal component analysis, value at risk, expected shortfall, G17, G32, C52, C53

## Abstract

This paper develops a method for assessing portfolio tail risk based on extreme value theory. The technique applies separate estimations of univariate series and allows for closed-form expressions for Value at Risk and Expected Shortfall. Its forecasting ability is tested on a portfolio of U.S. stocks. The in-sample goodness-of-fit tests indicate that the proposed approach is better suited for portfolio risk modeling under extreme market movements than comparable multivariate parametric methods. Backtesting across multiple quantiles demonstrates that the model cannot be rejected at any reasonable level of significance, even when periods of stress are included. Numerical simulations corroborate the empirical results.

## 1. Introduction

Events of the Global Financial Crisis, the Eurozone sovereign debt crisis, and the ongoing COVID-19 pandemics increase the investors’ awareness of the need to assess the risk of extreme losses and the necessity of protection against future market meltdowns. To quantify financial risks, investors and regulators commonly resort to risk measures based on quantiles of distributions of losses or returns, such as Value at Risk (VaR) or Expected Shortfall (ES). However, the most significant problem with conventional approaches to assessing the quantile-based risk measures is their poor performance in characterizing the “tail” behavior of the distribution of returns. Any model aiming to mitigate severe losses should specifically focus on the *tail risk*.

The importance of tail behavior in risk assessment has been emphasized by many authors, such as Poon et al. [1], Rodriguez [2], Ning [3], Okimoto [4], Ning [5], Christoffersen et al. [6], Rocco [7], Gkillas et al. [8], and Gkillas et al. [9], and many distributional assumptions have been introduced so far to improve risk management and asset pricing practices (see, for instance, [10,11]). Perhaps the most obvious alternative to the usual parametric approaches is the Extreme Value Theory (EVT) since it concentrates on extreme losses. McNeil [12], Bensalah [13], Smith [14], Nyström and Skoglund [15], Embrechts et al. [16], and Daníelsson [17], among others, provide excellent methodological overviews of EVT and discuss its applications in risk modeling. McNeil [18], Nyström and Skoglund [15], Harmantzis et al. [19], Marinelli et al. [20], and Lai and Wu [21] show that EVT-based models outperform VaR and ES estimates based on other analytical methods. The discrepancy becomes pronounced at more extreme quantiles.

The EVT approach seems like a natural choice for risk modeling: its implementation is relatively easy, and the method is based on a few assumptions required for the asymptotics to work. Although straightforward in the univariate case, EVT becomes of limited practical use when applied to a portfolio of financial instruments or several risk factors at once. Investors usually deal with many risk factors simultaneously, and hence an appropriate method requires a multivariate approach rather than a univariate one based on portfolio-level returns. As shown by Longin and Solnik [22], Campbell et al. [23], and many authors since, multivariate modeling is particularly vital during bear markets, when risk factors exhibit extreme co-movements. It also allows for risk attribution and additive decomposition of VaR, which is essential for risk management and economic capital allocation in financial institutions.

Defining portfolio-level dynamics of risk factors under extreme market conditions has so far been a daunting task. A seemingly obvious technique involves a multivariate version of the EVT based on the multidimensional limiting relations (see, for example, [14,24,25,26,27]). However, the model complexity increases faster than linearly with the number of risk factors in that case. Alternatively, the joint distribution of returns can be seen as a product of marginal distributions and a copula. McNeil and Frey [28] and Nyström and Skoglund [29], for example, describe the copula approach to the assessment of the extreme co-dependence structure of risk factors. The copula-based methods introduce an additional model risk, inherent in the assumption of a specific analytical form of the co-dependence function. In addition, they become quite intractable with an increase in dimensionality. Moreover, a typical copula method for multivariate EVT, such as the one described in Nyström and Skoglund [29], requires an additional simulation step to retrieve the residuals from the joint distribution, given the fitted marginals and parameters of the copula. The dimensionality problem in the multivariate setting can be tackled by vine pair copulas, as in Aas et al. [30], Chollete et al. [31], Yu et al. [32], or Trucíos et al. [33].

This paper introduces a multivariate EVT method for risk assessment based on separate estimations of the univariate model. Instead of estimating joint *n*-dimensional distributions (using multidimensional limiting relations, copulas, or otherwise), the method proposed here works with *n* orthogonal series that are approximately independent and identically distributed. These series are obtained from the eigenvectors of the joint return series, filtered to remove autocorrelation and heteroskedasticity. The filtering can be achieved, for instance, by assuming that the conditional covariance matrix follows a stationary *n*-dimensional model from the orthogonal GARCH family, as in Alexander [34] or van der Weide [35]. The tails of *n* independent univariate series of residuals are then fitted to a generalized Pareto distribution, allowing for closed-form expressions for VaR and ES. As an illustration, the technique is applied to a sequence of daily returns on stocks comprising the Dow Jones Industrial Average. For an out-of-the-sample analysis, daily VaR and ES estimates are compared to the actual portfolio losses over a more extended period, covering the extreme market co-movements during the last quarter of 2008. The results indicate that the method performs well in capturing extreme events jointly across the entire portfolio. The in-sample tests show that the proposed approach is better suited for portfolio risk modeling under extreme market movements than comparable multivariate parametric methods, such as orthogonal GARCH with normal or t-distribution. In addition, backtesting across multiple quantiles demonstrates that the method cannot be rejected at any reasonable level of significance, even for out-of-sample windows that contain stress periods.

This paper contributes to the literature in several important ways. To the best of our knowledge, it is the first method that extends the EVT to the multivariate case by combining the relative simplicity of univariate EVT and orthogonalization of return residuals. By doing so, we can capture tail correlations and extreme co-movements. The proposed method is universal, as it can be applied to any asset class, and it can incorporate any volatility model from the GARCH family.

The remainder of the paper is organized as follows: Section 2 presents the theoretical background behind the proposed multivariate EVT approach and the estimation methodology used in this paper. Section 3 describes the data, provides an example of VaR and ES estimation, and compares the method’s in-sample forecasting ability with other similar approaches. Section 4 illustrates the out-of-sample performance of the method through backtesting. Section 5 demonstrates the model performance on a set of simulated data. Concluding remarks are given in Section 6.

## 2. Theoretical Framework and Estimation Methodology

### 2.1. Theoretical Framework

Two important theorems are related to the univariate EVT. The first one shows that order statistics of independent and identically distributed (i.i.d.) random variables converge in distribution to the generalized extreme value (GEV) distribution [36,37]. The usual approach then is to fit the GEV distribution to the sequence of maxima of portfolio returns or their residuals, as described for example in Smith [14]. An alternative method is based on *exceedances over threshold*. The following theorem, first stated by Pickands [38], gives the asymptotic form of conditional distribution beyond a threshold:

**Theorem** **1.** *Let ${\left\{Z_{t}\right\}}_{t=1}^{T}$ be a set of T i.i.d. random variables with distribution function F. Define*

$$F_{u}\left(x\right):=P\left(Z_{t}\leq u+x\;|\;Z_{t}>u\right)=\frac{F(u+x)-F(u)}{1-F(u)},\;\;\;x\geq 0$$

*to be the distribution of exceedances of $Z_{t}$ over the threshold u. Let $z_{F}$ be the right endpoint of the distribution F, possibly a positive infinity. Then, if F is such that GEV distribution is well-defined, there are constants ξ∈R and $\beta :=\beta \left(u\right)\in R_{+}$ such that*

$$\lim_{u\to z_{F}}\underset{u<u+x<z_{F}}{\sup}\left|F_{u}\left(x\right)-G_{\xi ,\beta }\left(x\right)\right|=0,$$

*where*

(1) $$G_{\xi ,\beta }\left(x\right):=1-{\left(1+\xi \frac{x}{\beta }\right)}_{+}^{-1/\xi }$$

*is the generalized Pareto (GP) distribution, while the subscript + denotes the positive part of a function, i.e., $f_{+}\left(x\right)\equiv \max\left(f(x),0\right)$ for any function f.*

The challenges associated with implementation of EVT-based models are well known in the literature. Since the Theorem 1 defines a limiting distribution for the extreme returns, we need a sufficient number of observations *and* a large enough threshold *u*. There are different methods of making this choice, and some of them are examined in Bensalah [13] and Gabaix et al. [39]. In addition, the limit theorems hold only if the extreme observations $Z_{t}$ are i.i.d. Therefore, we cannot apply the results of univariate EVT to returns on financial assets directly without taking into account their autocorrelation and heteroskedasticity.

### 2.2. Estimation Methodology

The method works in several steps. We start from a multivariate series of returns in a portfolio consisting of *n* assets. Next, we apply orthogonal GARCH to obtain a set of random variables that are approximately i.i.d. Then, we fit the tails of *n* independent univariate residual series to a generalized Pareto distribution to obtain the quantiles for each component. Finally, we substitute these quantiles into a formula for VaR and ES. Section 2.2.1, Section 2.2.2, Section 2.2.3, Section 2.2.4, Section 2.2.5 and Section 2.2.6 describe the methodology in detail, while Section 2.2.7 provides its summary.

#### 2.2.1. Orthogonalization

The ultimate goal is to adapt the EVT approach to a portfolio consisting of *n* assets in order to capture their tail risk and their extreme co-movements. Before applying any of the results of EVT outlined in Section 2.1, we have to construct a set of cross-sectionally uncorrelated random variables. A natural choice is to work with the eigenvectors of the unconditional covariance matrix of returns.

**Definition** **1.** *Define $\varepsilon_{t}$ to be an n-dimensional random vector whose components $\varepsilon_{t,i}$ have zero mean for each i=1,2,…,n. Let $V_{\infty }=E\left(\varepsilon_{t}\varepsilon_{t}^{\prime }\right)$ be the n-by-n unconditional covariance matrix of $\varepsilon_{t}$ and P be the corresponding orthogonal matrix of normalized eigenvectors, where the prime symbol (′) denotes the transpose of a matrix. The eigenvalue decomposition of $V_{\infty }$ is given by*

$$V_{\infty }=P\Lambda P^{\prime },$$

*where* **Λ** *is a diagonal matrix of the eigenvalues of $V_{\infty }$, ordered by descending values, $\lambda_{1}\geq \lambda_{2}\geq \ldots \geq \lambda_{n}>0$. Further, let*

$$L:=P\Lambda^{1/2}.$$

*Then,*

(2) $$z_{t}=L^{-1}\varepsilon_{t},$$

*is called the vector of principal components of $\varepsilon_{t}$, for any t. The i-th element of the vector $z_{t}$ is called the i-th principal component of*$\varepsilon_{t}$.

Note that $E\left(z_{t}\right)=0$ and $\mathrm{var}\left(z_{t}\right)=1_{n}$, which follows from $E\left(\varepsilon_{t}\right)=0$ and $V_{\infty }=LL^{\prime }$, respectively. Hence, $z_{t}$ are cross-sectionally uncorrelated and each component has a unit variance. Since $\varepsilon_{t}=Lz_{t}$, each coordinate of $\varepsilon_{t}$ can be written as a linear combination of the principal components,

$$\varepsilon_{t,i}=\sum_{j=1}^{n}L_{ij}z_{t,j},\;\;\;i=1,2,\ldots ,n,$$

where $L_{ij}$ are the elements of L. The method can be easily adapted to fewer than *n* risk factors (see Appendix A).

An alternative linear transformation that can be used to separate multivariate returns into additive subcomponents is the independent component analysis. This technique has found its successful applications in finance: Back and Weigend [40] use it to extract the structure from stocks returns, while Moody and Wu [41] and Moody and Yang [42] apply it to foreign exchange rates. The main goal of independent component analysis is to minimize statistical dependence between the transformed vectors. Unlike principal component analysis, these vectors are neither orthogonal nor ranked by the variance they explain, and the linear transformation matrix analogous to L cannot be obtained in a closed form. Therefore, independent component analysis is not entirely suitable for the problem treated in this paper. A good comparison between the two methods can be found in Hyvärinen and Oja [43].

#### 2.2.2. Filtering

Orthogonalization transforms a cross-sectionally correlated series into a set of uncorrelated ones. We also have to filter out any autocorrelation and heteroskedasticity from the series. As the end-result, we will obtain sequences of conditional residuals that are orthogonal, and (approximately) serially uncorrelated and identically distributed.

Specifically, we can assume that, for each asset i=1,2,…,n, the series of log returns $y_{t,i}:=\ln\left(S_{t,i}/S_{t-1,i}\right)$, where $S_{t,i}$ denotes the price at time *t*, follows a process:

(3) $$y_{t,i}=\mu_{t,i}+\varepsilon_{t,i},$$

where $\mu_{t,i}$ is the conditional mean of $y_{t,i}$. Conditionally on the information available at t−1, the vector of residuals $\varepsilon_{t}:={\left[\varepsilon_{t,1}\;\;\varepsilon_{t,2}\;\;\ldots \;\;\varepsilon_{t,n}\right]}^{\prime }$ has a zero mean and a conditional covariance matrix $V_{t}$:

(4) $$\begin{array}{ccc} E\left(\varepsilon_{t}|F_{t-1}\right) & = & E\left(\varepsilon_{t}\right)={\left[0\;\;0\;\;\ldots \;\;0\right]}^{\prime }=:0, \end{array}$$

(5) $$\begin{array}{ccc} \mathrm{var}\left(\varepsilon_{t}|F_{t-1}\right) & = & E\left(\varepsilon_{t}\varepsilon_{t}^{\prime }|F_{t-1}\right)=:V_{t}, \end{array}$$

where, for any *t*, the matrix $V_{t}$ is positive definite and measurable with respect to the information set $F_{t-1}$, a σ-algebra generated by the past residuals $\left\{\varepsilon_{1},\;\varepsilon_{2},\;\ldots ,\;\varepsilon_{t-1}\right\}$.

To capture the volatility clustering effect, we can assume that the conditional covariance matrix follows a model from the GARCH family. The standard GARCH(*p*,*q*) is sufficient to capture most of the clustering, and—to some extent—excess kurtosis. The leverage effect can be taken into account, for example, by assuming that the conditional residuals follow an asymmetric distribution, such as skewed Student’s *t*. Alternatively, we can model the asymmetry explicitly in the equation followed by the conditional covariance matrix. Without losing generality of the proposed method, we can assume that the conditional covariance $V_{t}$ follows a multivariate asymmetric GARCH(*p*,*q*) of Glosten et al. [44], also known as the (multivariate) GJR-GARCH(*p*,*q*), or simply GJR(*p*,*q*):

(6) $$V_{t}=\Omega +\sum_{s=1}^{p}A_{s}E_{t-s}+\sum_{s=1}^{p}\Theta_{s}I_{t-s}E_{t-s}+\sum_{s=1}^{q}B_{s}V_{t-s},$$

where Ω, $A_{1},\;\ldots ,\;A_{p}$, $\Theta_{1},\;\ldots ,\;\Theta_{p}$, $B_{1},\;\ldots ,\;B_{q}$ are constant, positive semidefinite *n*-by-*n* matrices of parameters,

$$E_{t}:=\varepsilon_{t}\varepsilon_{t}^{\prime },$$

and

$$I_{t}:=\mathrm{diag}\left(\mathrm{sgn}{\left(-\varepsilon_{t,1}\right)}_{+}\;\mathrm{sgn}{\left(-\varepsilon_{t,2}\right)}_{+}\;\ldots \;\mathrm{sgn}{\left(-\varepsilon_{t,n}\right)}_{+}\right),$$

for any *t*. As usual, the matrices $A_{s}$ in (6) measure the extent to which volatility shocks in previous periods affect the current volatility, while $A_{s}+B_{s}$ measure the rate at which this effect fades away. The terms proportional to matrices $\Theta_{s}$ capture the impact of asymmetric return shocks to volatility. For any *t*, the unconditional covariance matrix of $\varepsilon_{t}$ is given by

$$V_{\infty }:={\left(1_{n}-\sum_{s=1}^{p}\left(A_{s}+\frac{1}{2}\Theta_{s}\right)-\sum_{s=1}^{q}B_{s}\right)}^{-1}\Omega ,$$

where $1_{n}$ denotes an *n*-by-*n* identity matrix. Covariance stationarity of the GJR(*p*,*q*) process (6) is assured by setting the matrix

$$1_{n}-\sum_{s=1}^{p}\left(A_{s}+\frac{1}{2}\Theta_{s}\right)-\sum_{s=1}^{q}B_{s}$$

to be positive definite. Note that the multivariate GJR process (6) contains the ordinary multivariate GARCH as its special case. There are also many plausible alternatives. For example, the APARCH model of Ding et al. [45] includes ARCH, GARCH, and GJR-GARCH models as special cases, as well as four other ARCH extensions. These four extensions are the TS-GARCH model of Taylor [46] and Schwert [47], Log-ARCH model of Geweke [48] and Pentula [49], N-ARCH model of Higgins and Bera [50], and T-ARCH model of Zakoïan [51]. Similarly, the EGARCH model of Nelson [52] can be used to capture the leverage effect in a slightly different manner. The method developed in this paper can be applied straightforwardly with any of the multivariate models from the GARCH family.

Cross-sectional correlations are reflected in the off-diagonal terms of matrices $V_{t}$ and $E_{t}$. This, in turn, makes the parameter matrices non-diagonal. In total, one would have to estimate (1+2p+q)(n+1)n/2 different parameters. Clearly, this number explodes as we increase the number of assets in the portfolio. However, this is only one aspect of the problem. The other is that we cannot apply the results of univariate EVT to conditional residuals $\varepsilon_{t,i}$ directly. However, we can switch to the orthonormal basis of principal components by applying the linear transformation (2) to the conditional residuals $\varepsilon_{t}$. In this basis, Equation (6) reads:

(7) $${\tilde{V}}_{t}=\tilde{\Omega }+\sum_{s=1}^{p}{\tilde{A}}_{s}{\tilde{E}}_{t-s}+\sum_{s=1}^{p}{\tilde{\Theta }}_{s}{\tilde{I}}_{t-s}{\tilde{E}}_{t-s}+\sum_{s=1}^{q}{\tilde{B}}_{s}{\tilde{V}}_{t-s},$$

where $\tilde{M}:=L^{-1}M{\left(L^{-1}\right)}^{\prime }$ for any square matrix M. In particular,

$${\tilde{E}}_{t}:=L^{-1}E_{t}{\left(L^{-1}\right)}^{\prime }=z_{t}z_{t}^{\prime }$$

and

$${\tilde{I}}_{t}:=L^{-1}I_{t}{\left(L^{-1}\right)}^{\prime }=\mathrm{diag}\left(\mathrm{sgn}{\left(-z_{t,1}\right)}_{+}\;\mathrm{sgn}{\left(-z_{t,2}\right)}_{+}\;\ldots \;\mathrm{sgn}{\left(-z_{t,n}\right)}_{+}\right).$$

Equation (4) implies that $E\left(z_{t}|F_{t-1}\right)=0$. Let

$${\tilde{V}}_{t}:=\mathrm{var}\left(z_{t}|F_{t-1}\right)=L^{-1}V_{t}{\left(L^{-1}\right)}^{\prime }$$

be the conditional covariance matrix of principal components. As in Alexander [34], it is reasonable to assume that the matrix ${\tilde{V}}_{t}$ is diagonal, since the eigenvectors $z_{t}$ are orthogonal. Then, the process given by Equation (7) can be estimated separately for each principal component. This gives a set of *n* independent scalar equations of the form

(8) $${\tilde{V}}_{t,i}={\tilde{\Omega }}_{i}+\sum_{s=1}^{p}{\tilde{A}}_{s,i}{\tilde{E}}_{t-s,i}+\sum_{s=1}^{p}{\tilde{\Theta }}_{s,i}{\tilde{I}}_{t-s,i}{\tilde{E}}_{t-s,i}+\sum_{s=1}^{q}{\tilde{B}}_{s,i}{\tilde{V}}_{t-s,i},$$

where, in general, ${\tilde{M}}_{i}:={\tilde{M}}_{ii}$ is the *i*-th diagonal element of the matrix $\tilde{M}$, *i* being 1,2,…,n for the first, second, *…*, *n*-th principal component, respectively.

Once we estimate the parameters $\tilde{\Omega },\;{\tilde{A}}_{1},\;\ldots ,\;{\tilde{A}}_{p},\;{\tilde{\Theta }}_{1},\;\ldots ,\;{\tilde{\Theta }}_{p},\;{\tilde{B}}_{1},\;\ldots ,\;{\tilde{B}}_{q}$, we can apply the inverse transformation

(9) $$V_{t}:=L{\tilde{V}}_{t}L^{\prime },$$

for t≥max{p,q}, to retrieve the series of conditional covariance matrices in the original basis of log returns. This allows us to forecast VaR and ES in a multivariate framework, and for an arbitrary portfolio.

#### 2.2.3. Estimating Independent Univariate Excess Distributions

Theorem 1 states that, for a large class of underlying excess distributions, the exceedances over threshold converge in distribution to GP as the sample size increases and the threshold is raised. Thus, the GP is the natural model for the unknown excess distribution above the threshold *u*, and we may conjecture that

(10) $$F_{u}\left(x\right)=G_{\xi ,\beta }\left(x\right),$$

for any *x* satisfying $0\leq x<z_{F}-u$. Assuming that we have a set of realizations for the variable $Z_{t}$, we can choose a sensible threshold *u* and estimate parameters ξ and β. If there are $T_{u}$ out of a total of *T* observations that exceed the threshold, the GP distribution will be fitted to the $T_{u}$ exceedances. In the literature, several estimators have been used to fit the GP parameters. The most popular ones are the maximum likelihood (ML) and the Hill estimator. The ML estimator assumes that, if the tail under consideration strictly follows a GP distribution, the likelihood function can be written in a closed-form. The estimators of the parameters ξ and β are then obtained using the standard ML approach. Provided that ξ>−1/2, the ML estimator of the parameters is consistent and asymptotically normal as the length of the series tends to infinity. On the other hand, the Hill estimator is based on a combination of the ML method and a semi-parametric result, which describes the scaling of *F* when ξ>0 (see, for instance, [53]). Here, we will use the ML estimator due to its performance and universality, as discussed in Nyström and Skoglund [15].

#### 2.2.4. Tails of Univariate Distributions

By combining Theorem 1 and Equation (10), we can write

$$F\left(z\right)=\left(1-F(u)\right)G_{\xi ,\beta }\left(z-u\right)+F\left(u\right),$$

for z>u. The only additional element we require to construct a tail estimator is F(u). The simplest choice would be to use the non-parametric method and take the obvious empirical estimator, $\hat{F}\left(u\right)=1-T_{u}/T$. Alternatively, we can find $T_{u}$ that is closest to a predetermined F(u). Thus, for example, in a sample of T=1000 observations, $\hat{F}\left(u\right)=0.90$ will correspond to $T_{u}=100$. The threshold is then set to $u=Z_{900}$, if ${\left\{Z_{t}\right\}}_{t=1}^{T}$ are ordered in an increasing way.

Combining the empirical estimate $\hat{F}\left(u\right)$ with the ML estimates of the GP parameters, we obtain the tail estimator:

(11) $$\hat{F}\left(z\right)=1-\frac{T_{u}}{T}{\left(1+\hat{\xi }\frac{z-u}{\hat{\beta }}\right)}^{-1/\hat{\xi }},\;\;z>u.$$

Note that, when the scale parameter β tends to infinity, $G_{\xi ,\beta }\left(\cdot \right)$ vanishes and the tail estimator converges to the empirical one for any *z*. Thus, the tail estimator in (11) can be viewed as the non-parametric estimator augmented by the tail behavior captured by the GP distribution.

#### 2.2.5. Estimating Univariate Var and Es

We formally define VaR in the following way.

**Definition** **2.** *Let ${\left\{Z_{t}\right\}}_{t=1}^{T}$ be a set of i.i.d. random variables with distribution function $F\left(z\right):=P\left(Z_{t}\leq z\right)$ for any i. Value at Risk is the α quantile of the distribution F:*

$${\mathrm{VaR}}_{\alpha }:=F^{-1}\left(\alpha \right),$$

*where α∈(0,1) and $F^{-1}$ is the inverse of F.*

The usual critique of VaR as a risk measure is that it is not coherent and ignores the structure of losses beyond a specific quantile. (See, for example, Artzner et al. [54,55], Acerbi and Tasche [56,57] and Szego [58].) In order to overcome these drawbacks, Expected Shortfall (ES) is often used as an obvious alternative. It is defined as the conditional expectation of loss that surpasses a fixed level of VaR. Formally, under the same assumptions as in Definition 2, we can define ES as:

$${\mathrm{ES}}_{\alpha }:=E\left[Z_{t}|Z_{t}>{\mathrm{VaR}}_{\alpha }\right].$$

As such, ES takes into account tail risk and satisfies the sub-additivity property, which assures its coherence as a risk measure.

Let $u_{+}\equiv u$ be the upper-tail threshold, and let the lower-tail threshold $u_{-}$ be defined symmetrically, that is, by $F\left(u_{-}\right)=1-F\left(u_{+}\right)$. Then, for a given upper-tail probability $\alpha_{+}>F\left(u_{+}\right)$ or a given lower-tail probability $\alpha_{-}<F\left(u_{-}\right)$, the general form of the VaR estimate is

(12) $$\begin{array}{ccc} {\hat{\mathrm{VaR}}}_{\alpha_{+}} & = & F^{-1}\left(\alpha_{+}\right)\;=\;u_{+}+\frac{{\hat{\beta }}_{+}}{{\hat{\xi }}_{+}}\left[{\left(\frac{T_{u_{+}}\left(1-\alpha_{+}\right)}{T}\right)}^{{\hat{\xi }}_{+}}-1\right], \end{array}$$

(13) $$\begin{array}{ccc} {\hat{\mathrm{VaR}}}_{\alpha_{-}} & = & F^{-1}\left(\alpha_{-}\right)\;=\;u_{-}+\frac{{\hat{\beta }}_{-}}{{\hat{\xi }}_{-}}\left[{\left(\frac{T_{u_{-}}\alpha_{-}}{T}\right)}^{{\hat{\xi }}_{-}}-1\right], \end{array}$$

where the subscript + (−) refers to parameters in the upper (lower) tail. Similarly, the general form of the ES estimate is

(14) $${\hat{\mathrm{ES}}}_{\alpha_{\pm }}=\frac{1}{1-{\hat{\xi }}_{\pm }}\left({\hat{\mathrm{VaR}}}_{\alpha_{\pm }}+{\hat{\beta }}_{\pm }-{\hat{\xi }}_{\pm }u_{\pm }\right).$$

(see, for example, [12]). As the tail becomes heavier, the tail index increases (equivalently, ${\hat{\xi }}_{\pm }\to 0$), and ES becomes progressively greater than VaR.

#### 2.2.6. Portfolio-Level VaR and ES

Given the upper- and lower-tail quantiles $\alpha_{\pm }$, the quantile of *i*-th principal component *h* time steps ahead is given by

(15) $$z_{\tau ,i}^{\pm }=F_{i}^{-1}\left(\alpha_{\pm }\right)\sqrt{{\tilde{V}}_{\tau ,i}},$$

where τ=T+h and $F_{i}^{-1}\left(\cdot \right)$ is the inverse of the univariate probability function for the set of principal components ${\left\{z_{t,i}\right\}}_{t=1}^{T}$, given by Equations (12) and (13).

Our final goal is to estimate VaR and ES for a portfolio of *n* assets. Denote by a the vector of portfolio positions, in monetary units. This, among other things, facilitates the treatment of portfolios with short positions. Then, *h*-steps-ahead portfolio VaR is given by

(16) $${\mathrm{VaR}}_{\alpha_{\pm }}=a^{\prime }\mu_{\tau }\pm \sqrt{a^{\prime }Q_{\tau }^{\pm }a},$$

where $Q_{\tau }^{\pm }$ is a real symmetric matrix that can be decomposed as

$$\begin{array}{ccc} Q_{\tau }^{\pm } & := & LD_{\tau }^{\pm }{\left(LD_{\tau }^{\pm }\right)}^{\prime }\\ D_{\tau }^{\pm } & := & \mathrm{diag}\left(z_{\tau ,1}^{\pm }\;z_{\tau ,2}^{\pm }\;\ldots \;z_{\tau ,n}^{\pm }\right). \end{array}$$

The intuition behind Formula (16) is fairly simple. The first term, $a^{\prime }\mu_{\tau }$, represents the forecast of the expected portfolio return. The second term is the upper- or lower-tail bound of the confidence interval for total portfolio return. It is obtained in three steps: first, we use (15) to calculate the quantiles $z_{\tau ,i}^{\pm }$, i.e., individual contribution to VaR for each principal component, and stack them into a diagonal matrix $D_{\tau }^{\pm }$; second, we use L to transform this matrix back to the original basis of log returns; finally, we take a square root of the matrix ”sandwich” consisting of the vector of portfolio holdings a and transformed quantiles $Q_{\tau }^{\pm }$ to obtain the bounds of confidence intervals around the expected return. If all principal components happen to be identically distributed (which is unlikely, even if all come from the same family of distribution functions), then the square-root term is simply the α-quantile of this distribution times the *h*-steps-ahead forecast of the portfolio variance.

A similar rationale can be applied to derive the forecast of portfolio ES:

(17) $${\mathrm{ES}}_{\alpha_{\pm }}=a^{\prime }\mu_{\tau }\pm \sqrt{a^{\prime }R_{\tau }^{\pm }a},$$

where, by analogy with Equations (14)–(16),

$$\begin{array}{ccc} R_{\tau }^{\pm } & := & L\Delta_{\tau }^{\pm }{\left(L\Delta_{\tau }^{\pm }\right)}^{\prime }\\ \Delta_{\tau }^{\pm } & := & \mathrm{diag}\left(\zeta_{\tau ,1}^{\pm }\;\zeta_{\tau ,2}^{\pm }\;\ldots \;\zeta_{\tau ,n}^{\pm }\right),\\ \zeta_{\tau ,i}^{\pm } & = & \frac{1}{1-\xi_{\pm }}\left(F_{i}^{-1}\left(\alpha_{\pm }\right)+\beta_{\pm }-\xi_{\pm }u_{\pm }\right)\sqrt{{\tilde{V}}_{\tau ,i}}. \end{array}$$

#### 2.2.7. Summary of the Methodology

We summarize the methodology through the following procedure:

**Step 1.** Start by performing the orthogonalization on portfolio returns. In other words, use the eigenvalue decomposition of the unconditional covariance matrix $V_{\infty }$ of return residuals $\varepsilon_{t}$ to obtain the matrix L following Definition 1. Then, use Equation (2) to obtain the set of orthogonal vectors $z_{t}$. This step assures cross-sectional independence and allows working with univariate series.

**Step 2.** Filter out the heteroskedasticity on each component from step 1 to obtain serially i.i.d. random variables. In other words, use the set of orthogonal vectors $z_{t}$ from step 1, calculate ${\tilde{E}}_{t}=z_{t}z_{t}^{\prime }$ and run a GARCH-like process such as one given by Equation (8) on ${\tilde{E}}_{t}$. This results in a set of univariate estimates of the conditional variance ${\tilde{V}}_{t,i}$. This step is a prerequisite for using the univariate EVT.

**Step 3.** Use the estimator from Equation (11) to fit the tails of each of the independent series from step 2. This gives the parameters of GP distribution.

**Step 4.** Substitute the parameters from step 3 into Equation (12) or (13). This step allows us to calculate the quantile for each component, Equation (15).

**Step 5.** Substitute the quantiles from step 4 into the closed-form expressions from Equations (16) and (17) to obtain portfolio VaR and ES.

Steps 1 and 2 are analogous to the orthogonal GARCH of Alexander [34]. Steps 3 and 4 are typical for a univariate EVT approach. A combination of steps 1–4 with step 5 is essential for the multivariate EVT approach.

As discussed, the method is based only on a few assumptions that allow the exploitation of asymptotical properties. From Theorem 1, we can see that it is sufficient to have a set of independent univariate random variables ${\left\{Z_{t}\right\}}_{t=1}^{T}$ drawn from the same distribution function *F*. We can obtain the set of univariate random variables by merely applying a simple eigenvalue decomposition of the unconditional covariance matrix $V_{\infty }$ in step 1. This step is well defined as long as the original multivariate series has a finite unconditional covariance matrix, a reasonable assumption for the financial time series. The set of i.i.d. random variables having the same distribution function *F* can be obtained through a GARCH-like filter in step 2. The filter can be applied provided that the covariance stationarity condition has been met. This condition is merely a restriction on the model parameters. Other assumptions are not required for the method to work since it relies on the transformed random variables’ asymptotic property.

## 3. Illustration of the Method

**Figure 1:**
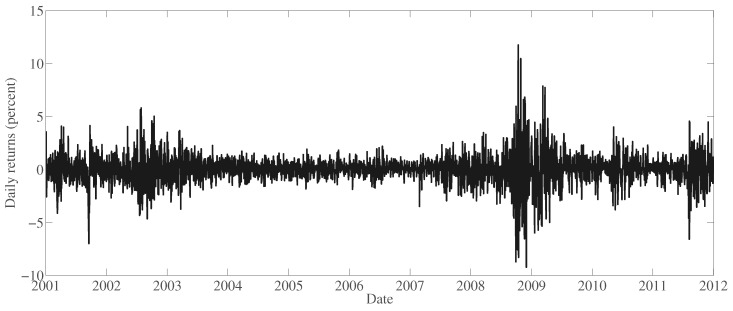
Daily returns of an equally-weighted portfolio of Dow 30 companies.

The empirical results that follow are based on daily closing prices of the thirty stocks that constituted the Dow Jones Industrial Average at the end of 2011, gathered from Thomson Reuters Datastream. The prices are adjusted for stock splits and dividend payments. The observation period covers all trading days between and, including 2 January 2001, and 30 December 2011, there is a total of 2766 returns per stock. Figure 1 displays the returns of an equally-weighted portfolio, which will be used in the later examples.

Following the procedure described in Section 2.2, we first estimate the upper- and the lower-tail parameters of the univariate GP distribution, $\xi_{\pm }$ and $\beta_{\pm }$, for each of the principal components. The upper and lower thresholds, $u_{+}$ and $u_{-}$, are determined by $F(u_{+})=0.90$ and $F(u_{-})=0.10$, respectively. (g choice of threshold $u_{\pm }$ is of minor importance for this illustration. For instance, using $F(u_{-})=0.05$ instead of 0.10 at α=0.001 changes the level of VaR forecast by less than a percent in relative terms.) The lower-tail VaR and ES forecasts can be found easily from Equations (16) and (17). The conditional covariance matrix ${\tilde{V}}_{\tau ,i}$ in (15) is obtained using the steps described in Equations (6)–(8). Here, we illustrate this procedure, assuming that the conditional covariance matrix in Equation (6) follows a multivariate GJR(1,1) process, i.e., p=q=1, or its multivariate GARCH(1,1) restriction, in which $\Theta_{s}\equiv 0$. Table 1 shows ten-day VaR and ES forecasts for several confidence levels reported as a percentage of the total portfolio value. (We choose the convention that the negative values obtained in Equation (15) correspond to losses.)

We can compare different multivariate volatility models in terms of their ability to fit the conditional distributions of principal components. Here, we use three different distributional assumptions: normal, Student’s *t*, and GP. The results for normal and Student’s *t*-distribution are calculated from the usual procedure of Alexander [34]. The GP results are based on our multivariate EVT method, as described in Section 2. The results are summarized in Table 2. The second column of Table 2 shows the cumulative $R^{2}$, i.e., the fraction of variance explained by each of the thirty eigenvectors. The remaining columns contain Kolmogorov–Smirnov test statistics for the univariate probability distribution of standardized eigenvectors $z_{t,i}$. The methods based on multivariate EVT appear to be the only ones that cannot be rejected at any reasonable significance level for all thirty eigenvectors—everything else being equal. Due to the fat tails of returns, conditional normality can be rejected for almost all eigenvectors. The assumption of Student’s *t* conditional distribution can be safely rejected for the first principal component, which is the one that explains almost half of the variations of returns in this sample. It is also rejected at more restrictive levels of significance for the remaining eigenvectors.

**Figure 2:**
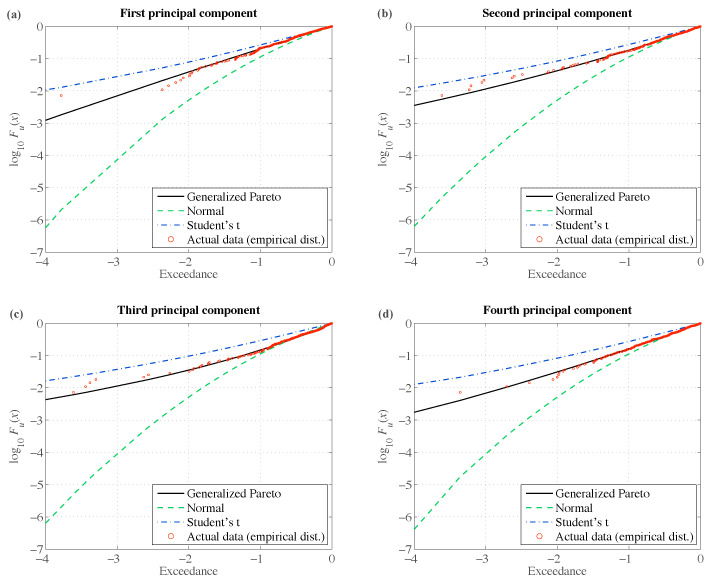
Distribution of exceedances in the lower tail of standardized residuals (log scale): (**a**) first principal component; (**b**) second principal component; (**c**) third principal component; (**d**) fourth principal component.

To visualize the model’s ability to fit the lower tail, we can plot the distribution of exceedances $F_{u}\left(x\right)$. Figure 2 shows $\log_{10}F_{u}\left(x\right)$ for the first four principal components, which jointly contribute to more than two-thirds of variations in portfolio returns. Each graph compares the empirical distribution function tails and three different distribution functions obtained from the orthogonal-GJR(1,1) residuals. The fitted GP distribution $G_{\xi ,\beta }\left(\cdot \right)$ is obtained from the model proposed in this paper. Consistently with Table 2, it provides the best fit to the actual data.

## 4. Out-of-Sample Performance

To analyze the out-of-sample performance of the proposed method, we use a 1000-day rolling-window sequence of one-day VaR forecasts and compare them to the corresponding actual returns of the equally-weighted portfolio for each day. In other words, we use the sequence of returns between 3 January 2001, and 14 January 2008, to obtain the VaR forecast for 15 January 2008. Then, we shift the window by one day and use the sequence of returns between 4 January 2001, and 15 January 2008, to obtain the VaR forecast for 16 January 2008, and so on, until the last sequence becomes the one corresponding to the period between 28 December 2004, and 29 December 2011, which is used to obtain the VaR forecast for 30 December 2011. We use five different VaR quantiles (α=0.001, 0.005, 0.010, 0.050, 0.100) and perform a backtesting procedure on the same models as in Section 3: orthogonal multivariate GARCH(1,1) and GJR(1,1) processes, under three distributional assumptions (normal, Student’s *t*, and GP).

Table 3 compares the actual and expected number of VaR violations for each quantile. We may notice that the number of violations is above the expected value, indicating that the models, on average, underestimate the tail risk. The assumption of conditional normality displays far too many exceptions in the extremes. The models based on the multivariate EVT method with GP distribution have violations whose number is closest to the expected.

We further follow Campbell [59] and apply a formal backtesting procedure that assesses the forecasting ability of each of the methods across multiple VaR quantiles simultaneously, separated into *K* bins on the unit interval. The test is based on the usual Pearson’s statistic,

(18) $$Q:=\sum_{k=1}^{K}\frac{{\left(N_{k}^{\mathrm{obs}}-N_{k}^{\exp}\right)}^{2}}{N_{k}^{\exp}},$$

where $N_{k}^{\mathrm{obs}}$ and $N_{k}^{\exp}$ are, respectively, the observed and the expected number of violations in the *k*-th bin. The *Q* statistic converges in distribution to a $\chi^{2}$ with K−1 degrees of freedom. The results of the test are summarized in Table 4 for the set of bins that correspond to the five VaR quantiles chosen, i.e., α∈[0.000,0.001)∪[0.001,0.005)∪[0.005,0.010)∪[0.010,0.050)∪[0.050,0.100)∪[0.100,1.000].

Table 4 indicates that the proposed multivariate EVT method cannot be rejected whenever the comparable methods can. For example, if the orthogonal multivariate GJR process is used, the model based on conditionally normal residuals can be rejected at 5% significance, but the models based on conditionally *t*-distributed residuals and the multivariate EVT method cannot. However, if the orthogonal multivariate GARCH process is used, then, at 5% significance, the models based on conditionally normal and *t*-distributed residuals can both be rejected, while the multivariate EVT method cannot. Similar results are obtained with Christoffersen [60] conditional coverage test across separate quantiles (available upon request). In Appendix B, we illustrate the method using the exchange-rate data. We obtain results similar to those shown in Table 4.

## 5. Numerical Simulations

To demonstrate the proposed method’s ability to incorporate various data patterns, we will apply it to simulated data in this section. To this end, we will simulate co-dependent multivariate series using different copulas: a Gaussian copula with the ‘’ordinary” (i.e., Pearson’s) linear correlation, a Gaussian copula with Kendall’s τ correlation, a *t* copula with Kendall’s τ correlation, and three Archimedean copulas—Clayton, Frank, and Gumbel. The copula parameters will be calibrated using the stock returns from Section 3. We will also use the stock data to obtain the marginal distributions, applying kernel smoothing on the empirical distribution functions. For the Gaussian copulas and the *t* copula, we apply the full 30-dimensional multivariate structure. Since the applied Archimedean copulas are bivariate, we use two equally-weighted sub-portfolios containing 15 return series each. For every copula, we simulate 10,000 data points in each series.

We again follow the procedure outlined in Section 2.2, with the same choice of thresholds. We then apply Equations (16) and (17) to estimate lower-tail VaR and ES. For the sake of tractability, we focus on the multivariate GJR(1,1) process for the conditional covariance matrix. Table 5 summarizes the ten-day VaR and ES forecasts for the same confidence levels as in Table 1, again reported as a percentage of the total portfolio value. We can notice that the numbers do not change drastically with respect to those reported in Table 1. They are also similar across different joint distributional assumptions.

**Figure 3:**
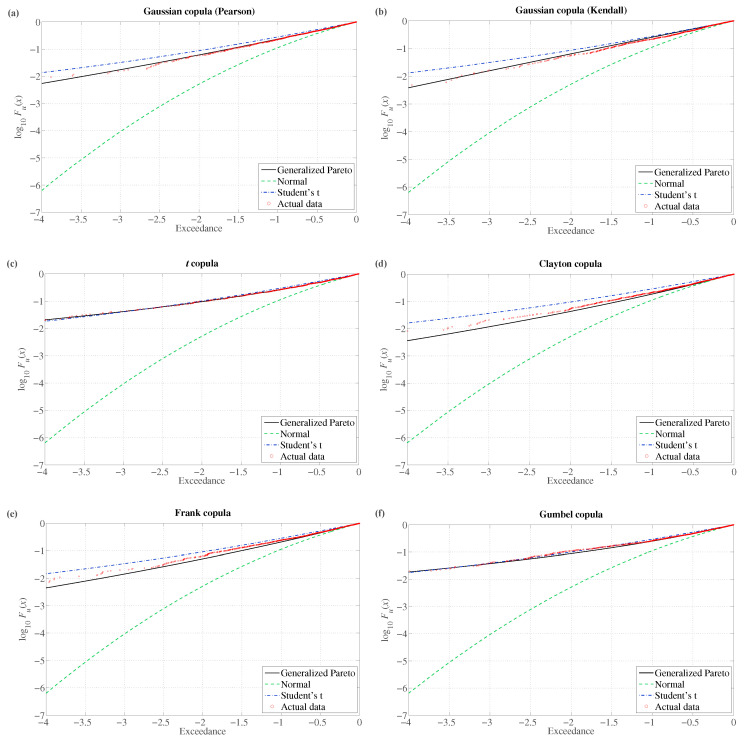
We show the $\log_{10}F_{u}\left(x\right)$ of the first principal component for each of the six copula types obtained from the orthogonal-GJR(1,1) residuals. (**a**) Gaussian copula (Pearson); (**b**) Gaussian copula (Kendall); (**c**) t copula; (**d**) Clayton copula; (**e**) Frank copula; (**f**) Gumbel copula.

The method’s flexibility in fitting the lower tail across various joint distributions of the data can be visualized in Figure 3. We show the $\log_{10}F_{u}\left(x\right)$ of the first principal component for each of the six copula types obtained from the orthogonal-GJR(1,1) residuals. The GP distribution $G_{\xi ,\beta }\left(\cdot \right)$ represents an excellent fit to the simulated data.

The out-of-sample performance of the proposed method on the simulated data can be assessed following the same idea as in Section 4. Again, we apply a 1000-day rolling-window sequence of one-day VaR forecasts. We use the same five VaR quantiles as in Section 4 and perform a backtesting procedure using the orthogonal multivariate GJR(1,1) processes, under six joint distributional assumptions (i.e., simulated copulas).

Table 6 shows the comparison between the actual and the expected number of VaR violations across quantiles. In all cases, the number of violations is very close to the expected value. We can see this more formally through Pearson’s *Q* test results, shown in Table 7. The proposed multivariate EVT method cannot be rejected for any of the six joint distributional assumptions used to obtain the simulated datasets.

## 6. Conclusions

This paper proposes a procedure for assessing portfolio tail risk based on Extreme Value Theory, without the necessity to use multivariate limiting relations. The method applies separate estimations of the univariate EVT model. It works with a set of orthogonal conditional residuals obtained from the principal components of the covariance matrix of returns. Autocorrelation, heteroskedasticity, and asymmetry inherent in the original return series can be removed by assuming a GARCH-type process for conditional variances of principal components. In this way, we can obtain a set of independent and identically distributed random variables, which is a prerequisite for any univariate EVT approach. The tails of the univariate distributions have been modeled by a generalized Pareto distribution of peeks over the threshold, while the interiors are fitted with an empirical distribution function. The estimation of parameters can be obtained using maximum likelihood, keeping the method computationally as fast as its orthogonal-GARCH counterparts. The method also provides closed-form expressions for Value at Risk and Expected Shortfall.

As an illustration, the method has been applied to a sequence of daily stock returns of Dow 30 companies. In-the-sample tests show that the proposed approach is more reliable for modeling extreme market movements than comparable multivariate parametric methods, such as orthogonal GARCH-type models with normal or *t*-distribution. Backtesting on 1000 out-of-the-sample observations indicates that VaR forecasts obtained by the proposed method perform well across multiple quantiles: the values of test statistics suggest that the null hypothesis of an equal number of observed and predicted VaR violations could not be rejected for all relevant VaR confidence levels. The importance of these results is further highlighted because the backtesting period covers extreme market movements that had followed the events of the Global Financial Crisis.

Numerical simulations have corroborated the empirical results. Using six different joint distributional assumptions, where various copula families drive the co-dependence in the data, we show that the proposed multivariate EVT method represents an excellent fit for the simulated data tails. The out-of-the-sample performance has also been confirmed via simulations: the method cannot be rejected for any of the six joint distributional assumptions.

Even though EVT is a natural choice for tail risk modeling, its major shortcoming is the complexity of extension to the multivariate case. This paper proposes a way to overcome this problem by combining the simplicity of univariate EVT and orthogonal GARCH while capturing tail correlations and extreme co-movements. The proposed method can be applied to any asset class, and it is straightforward to modify its filtering process to include any volatility model from the GARCH family. Hence, it has many potential applications in volatility forecasting, asset allocation under VaR or ES constraints, risk management of complex portfolios, or assessing individual assets’ contributions to systemic risk.

## Figures and Tables

**Table 1:** Ten-day VaR and ES Forecast.

	Confidence Level, 1−α				
	0.900	0.950	0.990	0.995	0.999
mv GARCH – GP					
VaR	5.11	6.77	10.81	12.63	17.08
ES	7.57	9.30	13.52	15.42	20.05
mv GJR – GP					
VaR	4.69	6.33	10.08	11.67	15.28
ES	7.04	8.66	12.35	13.90	17.46

**Table 2:** Model comparison: Kolmogorov–Smirnov statistics across eigenvectors.

eig.	Cumulative $R^{2}$	mv GARCH			mv GJR		
		Normal	Student	GP	Normal	Student	GP
1	0.4913	0.0470 ***	0.0367 ***	0.0053	0.0419 ***	0.0364 ***	0.0052
2	0.5609	0.0403 ***	0.0238 *	0.0060	0.0410 ***	0.0247 *	0.0040
3	0.6162	0.0362 ***	0.0177	0.0051	0.0363 ***	0.0280 **	0.0047
4	0.6523	0.0335 ***	0.0148	0.0041	0.0334 ***	0.0144	0.0044
5	0.6792	0.0396 ***	0.0126	0.0047	0.0388 ***	0.0125	0.0046
6	0.7053	0.0335 ***	0.0181	0.0061	0.0338 ***	0.0176	0.0073
7	0.7286	0.0571 ***	0.0126	0.0044	0.0571 ***	0.0143	0.0047
8	0.7489	0.0425 ***	0.0116	0.0070	0.0408 ***	0.0115	0.0062
9	0.7677	0.0302 **	0.0134	0.0045	0.0296 **	0.0139	0.0050
10	0.7862	0.0261 **	0.0170	0.0047	0.0260 **	0.0172	0.0046
11	0.8042	0.0308 **	0.0146	0.0059	0.0307 **	0.0147	0.0063
12	0.8214	0.0261 **	0.0162	0.0043	0.0264 **	0.0162	0.0041
13	0.8374	0.0310 ***	0.0167	0.0058	0.0295 **	0.0167	0.0063
14	0.8525	0.0320 ***	0.0180	0.0074	0.0316 ***	0.0177	0.0067
15	0.8668	0.0229	0.0194	0.0045	0.0225	0.0194	0.0052
16	0.8808	0.0277 **	0.0157	0.0053	0.0278 **	0.0156	0.0049
17	0.8939	0.0363 ***	0.0131	0.0041	0.0360 ***	0.0133	0.0039
18	0.9066	0.0302 **	0.0144	0.0052	0.0301 **	0.0134	0.0049
19	0.9188	0.0456 ***	0.0196	0.0050	0.0441 ***	0.0149	0.0041
20	0.9296	0.0347 ***	0.0240 *	0.0053	0.0365 ***	0.0149	0.0042
21	0.9396	0.0574 ***	0.0110	0.0058	0.0572 ***	0.0114	0.0056
22	0.9488	0.0405 ***	0.0168	0.0044	0.0405 ***	0.0164	0.0042
23	0.9576	0.0356 ***	0.0148	0.0035	0.0357 ***	0.0150	0.0040
24	0.9657	0.0266 **	0.0177	0.0076	0.0264 **	0.0173	0.0072
25	0.9736	0.0272 **	0.0140	0.0040	0.0280 **	0.0143	0.0040
26	0.9804	0.0519 ***	0.0097	0.0036	0.0519 ***	0.0107	0.0036
27	0.9863	0.0318 ***	0.0139	0.0052	0.0321 ***	0.0140	0.0043
28	0.9919	0.0362 ***	0.0196	0.0046	0.0364 ***	0.0196	0.0047
29	0.9972	0.0400 ***	0.0152	0.0062	0.0400 ***	0.0160	0.0065
30	1.0000	0.0326 ***	0.0136	0.0040	0.0327 ***	0.0140	0.0048

*—*p*-value <0.10; **—*p*-value <0.05; ***—*p*-value <0.01.

**Table 3:** Actual and expected number of lower-tail VaR violations for an equally-weighted portfolio of 30 stocks.

Method	Confidence Level, 1−α				
	0.900	0.950	0.990	0.995	0.999
mv GARCH					
Normal	109	69	24	16	7
Student	118	71	17	10	3
GP	117	68	15	8	3
mv GJR					
Normal	107	64	25	15	8
Student	117	65	19	10	1
GP	115	62	14	8	0
Expected	100	50	10	5	1

**Table 4:** Pearson’s *Q* test statistics and corresponding *p*-values for an equally-weighted portfolio of 30 stocks.

Method	*Q*	*p*-Value
mv GARCH		
Normal	46.77	0.000
Student	12.49	0.029
GP	9.62	0.087
mv GJR		
Normal	57.31	0.000
Student	10.75	0.057
GP	7.23	0.204

**Table 5:** Ten-day VaR and ES Forecast (simulated data).

	Confidence Level, 1−α				
	0.900	0.950	0.990	0.995	0.999
Gaussian copula (Pearson)					
VaR	5.07	7.19	12.29	14.58	20.08
ES	8.19	10.38	15.66	18.02	23.71
Gaussian copula (Kendall)					
VaR	5.26	7.41	12.34	11.67	19.25
ES	8.34	10.47	15.35	17.42	22.19
*t* copula					
VaR	5.01	7.17	13.67	17.28	28.14
ES	8.72	11.49	19.86	24.50	38.47
Clayton copula					
VaR	3.85	5.50	9.75	11.76	16.93
ES	6.38	8.19	12.83	15.04	20.68
Frank copula					
VaR	4.24	6.05	10.63	12.78	18.21
ES	6.99	8.94	13.89	16.21	22.07
Gumbel copula					
VaR	3.97	6.02	12.25	15.73	26.31
ES	7.51	10.18	18.97	22.79	36.52

**Table 6:** Actual and expected number of lower-tail VaR violations for an equally-weighted portfolio (simulated data).

Method	Confidence Level, 1−α				
	0.900	0.950	0.990	0.995	0.999
Gaussian copula (Pearson)	110	62	13	5	2
Gaussian copula (Kendall)	109	64	16	7	1
*t* copula	104	58	8	4	0
Clayton copula	110	52	12	6	0
Frank copula	100	45	10	8	1
Gumbel copula	101	51	12	8	1
Expected	100	50	10	5	1

**Table 7:** Pearson’s *Q* test statistics and corresponding *p*-values for an equally-weighted portfolio (simulated data).

Method	*Q*	*p*-Value
Gaussian copula (Pearson)	5.26	0.384
Gaussian copula (Kendall)	6.39	0.270
*t* copula	4.04	0.544
Clayton copula	3.59	0.610
Frank copula	5.18	0.394
Gumbel copula	2.48	0.780

**Figure A1:**
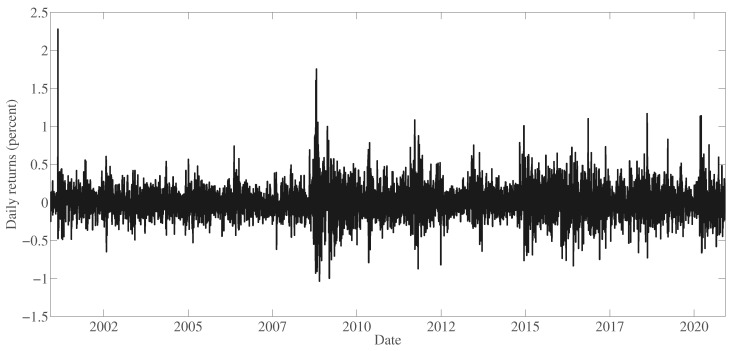
Daily returns of an equally-weighted portfolio of 20 major currencies against USD.

## Appendix A. Working with Fewer Than *n* Principal Components

Definition 1 implies that the fraction of total variation in $\varepsilon_{t}$ explained by the *j*-th principal component is given by

$$\frac{\lambda_{j}}{\sum_{k=1}^{n}\lambda_{l}}.$$

This property leads to another convenient feature of the principal component approach. Namely, if low-ranked components do not add much to the overall explained variance, which is often the case when the number of risk factors is relatively low, we can work with a reduced number of *m* principal components, where m<n. The first *m* components will then explain

$$\frac{\sum_{j=1}^{m}\lambda_{j}}{\sum_{k=1}^{n}\lambda_{l}}≲1$$

of the variation in $\varepsilon_{t}$. In that case, L is replaced by an *n*-by-*m* matrix $L_{m}$, where

(A1) $$L_{m}:=P_{m}\Lambda_{m}^{1/2},$$

$P_{m}$ is an *n*-by-*m* matrix of the first *m* normalized eigenvectors, and

$$\Lambda_{m}:=\mathrm{diag}\left(\lambda_{1}\;\lambda_{2}\;\ldots \;\lambda_{m}\right)$$

is a diagonal matrix of the first *m* eigenvalues. The *m*-dimensional vector of the first *m* principal components of $\varepsilon_{t}$ is then given by

(A2) $$z_{t}=L_{m}^{-1}\varepsilon_{t},$$

for any *t*, where $L_{m}^{-1}$ is the pseudo-inverse of $L_{m}$. It is straightforward to generalize the multivariate EVT approach presented in the paper to the case of m<n principal components. Using definition (A1), we can transform any *n*-by-*n* matrix M into the basis of the first *m* principal components via transformation

$$\tilde{M}:=L_{m}^{-1}M{\left(L_{m}^{-1}\right)}^{\prime },$$

obtaining an *m*-by-*m* matrix $\tilde{M}$. Equations (7), (8), (15), and (16) maintain the same form.

## Appendix B. Illustration Using Exchange-Rate Data

As an additional robustness check of our method, we present here the results for the exchange-rate data. On average, currency portfolios have lower rates of return than stocks but are significantly more volatile and notoriously fat-tailed. Here, we use daily data on 20 major currencies against the US Dollar (USD), retrieved from Refinitiv-Eikon. We used the following currencies: Australian Dollar, Brazilian Real, Canadian Dollar, Swiss Franc, Chinese Yuan, Euro, Pound Sterling, Hong Kong Dollar, Indian Rupee, Japanese Yen, South Korean Won, Mexican Peso, Norwegian Krone, New Zealand Dollar, Russian Ruble, Swedish Krona, Singapore Dollar, Turkish Lira, New Taiwan Dollar, and South African Rand. The data consists of middle exchange rates between 1 December 2000, and 1 December 2020, a total of 5217 returns per currency. Figure A1 shows the returns of an ‘’equally-weighted” portfolio, which we will apply in our analysis, analogous to Section 3.

We use a 2000-day rolling-window sequence of one-day VaR forecasts to analyze the out-of-sample performance on the exchange-rate data. As in Section 4, we compare these results to the corresponding actual returns of the equally-weighted portfolio for each day using the same five quantiles (α=0.001, 0.005, 0.010, 0.050, 0.100) and the same multivariate methods. The results are summarized in Table A1 and Table A2. Table A1 compares the actual and expected number of VaR violations for each quantile. The multivariate EVT method with GP distribution has the number of violations that is again closest to the expected. We can see this more formally from the backtesting results in Table A2. Again, the proposed multivariate EVT method cannot be rejected whenever the comparable methods can. This result indicates that the method is robust across different asset classes, observation periods, and backtesting horizons.

**Table A1:** Actual and expected number of lower-tail VaR violations for an equally-weighted portfolio of 20 major currencies against USD.

Method	Confidence Level, 1−α				
	0.900	0.950	0.990	0.995	0.999
mv GARCH					
Normal	264	169	53	34	20
Student	248	147	32	21	9
GP	217	115	25	13	2
mv GJR					
Normal	225	140	36	27	15
Student	251	145	31	22	8
GP	207	111	24	12	1
Expected	200	100	20	10	2

**Table A2:** Pearson’s *Q* test statistics and corresponding *p*-values for an equally-weighted portfolio of 20 major currencies against USD.

Method	*Q*	*p*-Value
mv GARCH		
Normal	193.33	0.000
Student	43.20	0.000
GP	2.98	0.704
mv GJR		
Normal	96.40	0.000
Student	38.85	0.000
GP	2.82	0.727
